# A Linear Periodized Resistance Training Program Is Effective at Reducing Depressive Symptoms but Not Anxiety in Females: A Pilot

**DOI:** 10.3390/jcm14030853

**Published:** 2025-01-28

**Authors:** Jason Sawyer, Matthew McPherson, Meghan Reuter, Paul A. Cacolice

**Affiliations:** 1 Department of Biological and Biomedical Sciences, Bryant University, Smithfield, RI 02917, USA; jsawyer7@bryant.edu; 2 Clinical Team, Early Medical, Austin, TX 78746, USA; mmymcpherson@gmail.com; 3 Athletics Department, University of Notre Dame, Notre Dame, IN 46556, USA; mreuter@nd.edu; 4 Department of Sports Medicine and Human Performance, Westfield State University, Westfield, MA 01086, USA

**Keywords:** anxiety, depression, generalized anxiety disorder, major depressive disorder, resistance training, strength training

## Abstract

**Background/Objectives**: The purpose of this study was to determine whether a periodized resistance training program would influence self-reported depression and anxiety scores in college-aged females. **Methods**: Eight participants participated in a six-week periodized resistance training program. The participants completed a 3–5 repetition maximum (3–5 RM) for the sumo deadlift (SDL), bench press (BP), barbell back squat (BBS), and standing shoulder press (SSP). These data were used to estimate the 1 repetition maximum (1RM), which in turn was used to develop the periodization program. Following baseline testing, participants participated in two full-body workouts per week for six weeks. Each individual was retested after they completed the 6-week program, performing 3–5 RM for the SDL, BP, BBS, and SSP. To determine symptoms of depression and anxiety, the Beck Depression Inventory (BDI) and Beck Anxiety Inventory (BAI) were distributed before and after participating in the resistance training program. A repeated measures 2 × 2 Analysis of Variance (ANOVA) was used to determine the effect of resistance training had on the outcome measures. **Results**: There was a significant (*p* = 0.011) decrease in BDI scores after the 6 weeks of resistance training. There was no statistically significant difference in the BAI scores (*p* = 0.106). There was no correlation between any individual exercise and the outcome scores. **Conclusions**: The results of the current study indicate that a periodized resistance training program is effective at reducing self-reported measures of depression using the BDI in college-aged females.

## 1. Introduction

The illnesses of depression and anxiety are common and costly, both to the individual and society. The American Psychiatric Association (APA) defines depression as a medical illness that causes feelings of sadness and/or apathy towards activities individuals once enjoyed, with the symptoms lasting longer than two weeks [1]. Other symptoms of depression include idiopathic hypersomnia, decreased energy levels, irritability, weight loss or gain, trouble concentrating, and suicide ideation [1]. Commonly, depression and anxiety are co-occurring, which increases the clinical severity of the diseases [2,3].

The conventional treatment for depression is a combination of pharmacological interventions, such as selective serotonin reuptake inhibitors (SSRI), and psychotherapy, including cognitive behavioral therapy [4]. The combination of these treatments has modest effect sizes and varies in patient response [5]. For example, approximately one third of individuals that engage in these treatments report no significant change in depressive symptoms [6,7]. Moreover, psychiatric medications may cause side effects, including nausea, diarrhea, loss of libido, sexual dysfunction, tiredness, and increase the risk for metabolic syndrome, diabetes, and heart problems [8]. Because of the modest effect size, varying response by individuals, differences in access to healthcare, and potential side effects of antidepressant medication, alternative methods of treating depressive symptoms must continue to be explored. Historically, various strategies have been attempted for both palliative and curative efforts. Among these are: light exposure, dietary and vitamin supplementation [9], mindfulness [10], and meditation [11], each with varying degrees of effectiveness. Aerobic and/or resistance training may be further means of decreasing depressive symptoms [9].

The positive benefits of aerobic exercise on depressive symptoms in various populations are well established [12]. In the clinically depressed, Morres [13] determined that aerobic exercise decreased self-reported feelings of depression. Other researchers determined that aerobic exercise is effective at decreasing feelings of depression in adolescents [14], adults [15], and older individuals [16]. Moreover, physical activity may have a protective effect against depressive symptoms [17].

While the evidence supporting the antidepressant effects of aerobic exercise is strong, there are conflicting results regarding the optimal exercise intensity for decreasing depressive symptoms. In college-aged individuals, Liu [18] concluded that there was greater reduction in depressive symptoms after performing high-intensity aerobic exercise compared to moderate- or low-intensity exercise. Conversely, Paolucci [19] investigated the influence of aerobic exercise intensity on depressive symptoms and determined that moderate-intensity exercise was more effective at decreasing depressive symptoms compared to high-intensity exercise. Finally, Meyer [20] concluded that light-, moderate-, and high-intensity exercise were equally as effective for reducing depressive symptoms in females. Examined in totality, the research indicates that aerobic exercise of any intensity may decrease depression, with a larger effect size observed with moderate-intensity aerobic exercise [19,21].

While abundant research on depression and aerobic exercise exists, there is less literature examining the effects of resistance training on depression. Furthermore, many studies examining the effects of resistance training and depression used an elderly population with disease. In a meta-analysis, Rossi [22] reported that out of 38 studies included, 26 were conducted with the elderly. The authors concluded that resistance training decreases depressive symptoms, and the effect size may be small to moderate [22]. Resistance training program variables, such as frequency and volume, may partially explain the effectiveness of the program in alleviating feelings of depression [22].

The Diagnostic and Statistical Manual (DSM-5) defines anxiety as excessive worry and apprehension that occurs more days than not for the previous 6 months [1]. The symptoms include restlessness, increased fatigability, irritability, difficulty concentrating, and sleep disturbances [1].

The anxiolytic effects of a single bout of exercise are well-established [23]. In a meta-analysis, researchers [24] determined that a single bout of exercise reduces reported symptoms of state anxiety in adults. Additionally, a single bout of resistance training exercise decreases state anxiety in college-aged individuals [25]. The evidence supporting the efficacy of exercise on trait anxiety is conflicting. Ligeza [26] concluded that bouts of acute aerobic exercise decrease trait anxiety in adolescents. Conversely, in a meta-analysis [27] of 25 random controlled trials on the effects of exercise on trait anxiety, 18 of them reported equivocal findings and/or no change in trait anxiety. The conflicting results may be partially explained by the exercise intensity used during the research studies, as high-intensity exercise may be more effective at reducing anxiety levels compared to low-intensity activity [28]. Considered in totality, the research suggests that exercise, particularly high intensity, decreases state anxiety in individuals [24,28].

Previous research has established the relationship between aerobic exercise and depression and anxiety [23,29]. However, the literature supporting the effect of resistance training on depression and anxiety symptoms is limited, particularly with the use of a linear periodized program. The purpose of this preliminary pilot investigation was to explore the association between a progressive resistance training program and measures of depression and anxiety. We hypothesized that resistance training would cause a decrease in feelings of depression and anxiety similar to those observed during aerobic exercise.

## 2. Material and Methods

Participants were recruited from a university counseling center, utilizing both referrals and recruitment flyers displayed in the campus fitness centers. Inclusion criteria included that participants were familiar with the resistance training exercises but had not been actively involved in a resistance training program during the previous six months. The study was conducted according to the guidelines of the Declaration of Helsinki and approved by the Westfield State University Institutional Review Board (IRB).

During the initial visit, participants completed a medical history questionnaire, a training history, a Beck Depression Inventory (BDI), and a Beck Anxiety Inventory (BAI). Written informed consent was obtained from all participants involved in the study. Participants were excluded from the research study if there was a known cardiovascular disease or if a musculoskeletal injury was present. Furthermore, participants were excluded if they had participated in a resistance training program during the previous six months. Other exclusion criteria included a clinical diagnosis of a depression or anxiety disorder or currently prescribed medication to treat depression or anxiety. During the resistance training program, participants were asked to refrain from performing aerobic exercise. Body weight and body composition were not measured during this investigation to minimize the impact of such tests on depression and anxiety [30,31,32].

Before the start of the testing session, the participants performed a warm-up consisting of dynamic stretches for the musculotendinous tissues directly impacted by the selected resistance training activities: hamstrings, quadriceps, gluteals, calves, deltoids, and of the anterior chest wall. Dynamic stretches were performed as these have been shown to reduce injury [33] and improve muscle activation [34], thus facilitating improved participant performance. After the warm-up, the participants completed 3–5 RM testing for the following exercises: bench press (BP), barbell back squat (BBS), standing shoulder press (SSP), and sumo deadlift (SDL) utilizing the protocol outlined by Haff and Triplet [35]. These specific training activities were selected as they each require multiple arthro-kinematic actions and musculotendinous utilization for successful completion. That is, these multiple joint motion activities increase the overall training intensity to the individual in the time constraints of the training session. The participant’s 1RM was then estimated using the Brzycki equation [36]. This equation is commonly used in the field of strength and conditioning and has been independently validated in various populations [37,38]. After completing the testing session, each participant was provided with an individualized 6-week linear periodization program. This program was reviewed with the participant prior to the initial training session.

For the next 6 weeks, the participants performed resistance training twice per week. All training sessions were supervised by a research assistant to ensure safety and compliance. The core lifts for the program were the BP, BBS, SSP, and SDL, and the initial intensity for these exercises was set at 75% of the estimated 1 RM and gradually increased to 90% by week 6. A detailed description of exercise intensity, repetitions, and sets is provided in Table 1. Each week, the intensity increased by 2–3%, with a corresponding decrease in repetitions. During each workout, 5 accessory exercises were included and participants completed 8–10 repetitions for 4 sets at a self-selected intensity. When the participants could complete all the repetitions for the 4 sets, the weight was increased by 2.2 kg. The inter-set rest period was 3 min throughout the entirety of the program. During the last session, the participants repeated the testing to determine 3–5 RM and 1 RM estimation. After completion of the final testing sessions, participants completed the BDI and BAI.

A repeated-measures 2 × 2 Analysis of Variance (ANOVA) was used to determine the effect of the resistance training program on measures of depression and anxiety. Pre- and post-estimated 1 RM for BP, BBS, SSP, and SDL were analyzed using a repeated-measures 2 × 4 ANOVA. Pre-and post-training values for depression, anxiety, BP, BBS, SSP, and SDL were entered into statistical analysis software (SPSS v24, IBM, Armonk, NY, USA). Values for the net difference between pre- and post-training of each variable were calculated. An alpha error probability value was set at *p* ≤ 0.05.

## 3. Results

Compliance during the program was 100%, with participants completing all exercise sessions with no adverse effects. The age range of the participants was 18–22 years old, with a mean of 20.4 years. All participants self-identified as Caucasian. The means and standard deviations of all measured variables are shown in Table 2. None of the participants reported receiving psychotherapy during the six weeks of training.

The number of participants in specific score ranges from pre- to post-intervention for BDI is listed in Table 3.

The number of participants in specific score ranges from pre- to post-intervention for BAI is listed in Table 4.

### 3.1. Strength Measures

BBS and SDL significantly increased from pre- to post-testing. There was no significant increase in BP and SSP from pre- to post-training.

### 3.2. Depression and Anxiety

The difference between pre-and post-training was significant for BDI scores (*p* = 0.011) but not BAI scores (*p* = 0.106). A 2 × 4 ANOVA was generated to ascertain statistical associations between each of the independent variables (BP, BBS, SSP, and SDL) and the dependent variable (depression). There were no statistically significant associations between any other independent variables (training activities) and the dependent variables (depression, anxiety) at *p* ≤ 0.05. From pre- to post-training, participants significantly increased BBS and SDL. There was no significant increase in BP and SSP from pre- to post-training.

## 4. Discussion

The purpose of this pilot investigation was to explore the effects of a linear periodized resistance training program on patient-reported outcome tool measures of depression and anxiety. The primary finding of this pilot study was that a 6-week resistance training program decreased outcome measures of depression in college-aged females. There was no change in self-reported anxiety scores before and after the exercise program.

Each exercise session was supervised by a research assistant. The research assistants in this investigation had completed an academic course of instruction for resistance training supervision instructed by one of the study authors. This supervision was direct for the duration of each training session for both safety and effectiveness [35]. In a meta-analysis, Chen [15] concluded that supervision during aerobic exercise programs is salient in determining the effectiveness of the program at reducing depressive symptoms, partially by decreasing the dropout rate among participants.

The observed gains in strength in a short training window should not be excessively surprising in the untrained population. The strength gains made in those with depression symptoms, however, should be of specific note. As previously stated, depression symptoms can cause apathy towards activities, decreased energy levels, irritability, and/or trouble concentrating [1]. The necessary degree of each of these is hyper-focused when performing high-intensity resistance training such as was utilized in this investigation. Likewise, the training regimen in this study was limited in duration and variability commonly seen with more extensive schemes, such as more complex periodization models.

Previous researchers [39,40,41] determined similar reductions in reported symptoms of depression. In a meta-analysis, O’Connor [41] concluded that strength training decreases depressive symptoms in individuals diagnosed with depression and those with fibromyalgia. Despite the effectiveness of strength training on depression, the researchers highlight issues with the research including variations in the strength training program prescribed. Doyne [42] compared the effects of 8 weeks of weightlifting versus running on depression in females and determined that both interventions reduced reported symptoms of depression. Similarly, Craft and Perna [40] reported that exercise decreases depressive symptoms without accompanying improvements in physical fitness, and Barbour [39] contends that despite “significant methodological limitations” in exercise and depression literature, exercise is effective at reducing symptoms of depression in older adults compared to waitlist, social contact controls, and antidepressant medication.

The heterogeneity of resistance training programs utilized during research studies makes determining minimal dose recommendations difficult. In a meta-analysis, Augustin [43] reported that resistance training programs included in research studies ranged from 8 weeks to 8 months. Others [44] determined that a 9-month resistance training program was effective at improving quality of life and reducing depressive symptoms in an elderly population. Fisher [45] reported that the minimal dose of resistance training for health benefits is ≤60 min, 2 d-wk^−1^. Interestingly, these authors [45] encourage medical professionals to “prescribe resistance training like a drug”. In a study examining the effects of resistance training on depression in the elderly, Singh [46] reported that 33% of participants continued performing strength exercises 26 months after the conclusion of the study. Importantly, the individuals who continued resistance training reported lower levels of self-reported symptoms of depression compared to those who stopped exercising [46].

The mechanisms by which resistance training reduces reported symptoms of depression have yet to be elucidated. Resistance training may improve self-esteem, which could influence depressive symptoms. Adolescents who engaged in a resistance training program reported increases in self-efficacy 6 months after starting the program [47]. Moreover, Kercher [48] determined that 16 weeks of resistance training improved self-efficacy in adults. O’Connor [41] referenced “at least 6 randomized trials” that concluded strength training alone led to improved self-esteem and may thus decrease reported feelings of depression. A linear periodized program allows for the tracking of volume, which could be a definite way to demonstrate progress. Furthermore, a linear periodized program is an effective way to increase muscular strength [49], motor ability, and possibly improve body composition [50]. The increases in strength and body composition as a result of a linear periodized resistance training program may, through improved self-esteem, lead to a reduction in depression. Furthermore, in the elderly population, the resultant increase in muscular strength and motor ability may increase the quality of life by allowing individuals to perform tasks that may not have been possible before [51,52,53,54].

One previously identified population at elevated risk of depression and anxiety are elite athletes. Female athletes are at elevated risk over their age-matched, non-athlete peers [55,56]. Experiencing an injury that prohibits participation in athletics further compounds this elevated risk [55]. Findings from the current investigation suggest that elite athletes participate in high-intensity resistance training as much as possible during their recovery from injury as a means to reduce the risk of depression and anxiety.

To determine the mechanisms by which exercise may decrease depressive symptoms, Pahlavani [57] examined the effects of aerobic exercise on neurotransmitter levels. They concluded that aerobic exercise increases key neurotransmitters such as serotonin, dopamine, GABA, and acetylcholine, positively influences neuromodulators, and decreases inflammatory markers. Akbarpour [58] reported that eight weeks of resistance training can increase dopamine and serotonin levels in males recovering from substance abuse disorder. Furthermore, brain derived neurotrophic factor (BDNF) levels are increased by 98% after a bout of resistance training compared to baseline [59]. A hallmark characteristic of depression is a decrease in neuroplasticity of the hippocampus [60]. Resistance training increases connectivity [61] and neuroplasticity [62] in the hippocampus.

Interestingly, anxiety outcome measures were unchanged in the current research. It is possible that exercise, specifically resistance training, is effective at decreasing state anxiety levels but has less effect on trait anxiety. In support of this theory, Córdoba-Grueso [63] determined that aerobic exercise in adolescents does not decrease the risk of anxiety disorders in adulthood. Participants in the current study completed the BAI a minimum of 24 h after their last training session, and therefore the anxiolytic effects of resistance training may be short-term. Bibeau [25] examined the effects of a single bout of resistance training of varying intensities and rest times on state anxiety scores in college-aged individuals. The researchers [25] determined that low-intensity resistance training had a greater benefit compared to higher intensities. During the current investigation, the multi-joint compound exercises were prescribed at an initial level of 75%. It is possible that this intensity is too high to reduce feelings of anxiety.

Ecologically valid resistance training aims to mimic authentic training programs commonly followed by lifters [64]. Compared to programs typically used in a laboratory setting, ecologically valid resistance training programs increase generalization. Gordon [64] determined that an ecologically valid resistance training program decreased anxiety symptoms in a subclinical, college-age population. They designed the resistance training program utilized in the study following the WHO and ACSM guidelines. These guidelines recommend that participants perform between 8 and 12 repetitions for exercises and increase the intensity of the exercises once 12 repetitions can be performed [65]. Although these guidelines may be adequate for the general population, there are repetition ranges (i.e., low ranges) excluded that may benefit athletes [66]. Specifically, Schoenfeld [66] reported that muscular strength is increased more by using high loads with low repetitions compared to lower loads with higher repetitions. This difference is important for individuals attempting to maximize muscular strength gains through resistance training.

Similar to depression, the mechanisms through which resistance training reduces anxiety have not been established. Deng [67] determined that college-aged individuals who engaged in an acute bout of resistance training reported increased feelings of life satisfaction and self-efficacy. They concluded that increasing life satisfaction and self-efficacy is “key” to reducing state anxiety. The physiological mechanisms by which exercise, including resistance training, may reduce anxiety are similar to those for depression, and include stabilizing the HPA axis to reduce stress and anxiety, altering the monoamine, opioid, and/or neurotrophic factors, or increasing neurogenesis in the hippocampus [68].

During the current study, body weight or body composition measurements were not administered. Previous researchers [32] defined anxiety about weight gain as a negative reaction to increasing body weight. While the impacts of professionals measuring body weight are unknown, the negative effects of self-weighing are well documented [31]. In a meta-analysis of 23 studies, researchers [31] determined that 13 studies reported negative associations between self-weighing and affect, body evaluation, and eating behavior. Furthermore, anxiety levels may increase for individuals who are now normal weight but have a history of being overweight or obese [32]. Perceived body weight may also increase depressive symptoms in college-aged females [30]. Given the evidence regarding mental health and body weight and to avoid causing any negative feelings including depression or anxiety, the exclusion of these tests was intentional.

A major limitation of this pilot study was the lack of a control group. In a systematic review, Arain [69] reported that more than 30% of pilots published in medical journals did not include a control group. A future, large-scale study should include a control group to determine the effects of a linear periodized resistance training program on depression and anxiety. Furthermore, future studies should incorporate means of assessing psychological well-being as well as potential changes in self-perception, self-esteem, and quality of life. Those who are depressed are less likely to participate in physical activity, resulting in a decrease in physiological as well as psychological health [70]; resistance training may have the dual benefit of improving both. Having positive self-perception and high self-esteem is associated with better mental health [41], and there are several aspects of heavy resistance training that could contribute to improvements in these markers. Moreover, the effects of co-diagnoses of other diseases, such as substance misuse disorder, should be assessed. Finally, future studies should include ways to determine continuation rates comparing resistance training to traditional pharmacological treatments, as the latter has been shown to have an early discontinuation rate of nearly 50% [71].

## 5. Conclusions

Abundant research supports that aerobic exercise reduces reported symptoms of depression in various populations, including those with major depressive disorder, adolescents, the elderly, and those with chronic diseases. Moreover, state anxiety may be reduced by aerobic exercise. Despite the overwhelming evidence in support of aerobic exercise in the treatment of depression and state anxiety, resistance training has been studied less extensively. Resistance training does decrease reported symptoms of depression and anxiety in the elderly and college-aged individuals. Most resistance training programs employed in research studies do include periodization typically used in the field of strength and conditioning. Furthermore, the effects of resistance training on subthreshold depressive symptoms remain unknown. This study suggests that a linear periodized resistance training is effective at lowering depressive symptoms in subthreshold, college-aged females but not trait levels of anxiety.

## Figures and Tables

**Table 1:** Description of exercise intensity, repetitions, and sets for the core lifts.

Week	Intensity for Core Lifts	Repetitions	Sets
1	75%	10	4
2	78%	8	4
3	80%	6	4
4	85%	5	4
5	87%	3	4
6	90%	2	4

**Table 2:** Means and Standard Deviations for Independent and Dependent Variables.

	Pre-Training			Post-Training	
	Mean	Standard Deviation		Mean	Standard Deviation
BP 1RM (kg)	32.759	10.745		36.287	10.242
BBS 1RM (kg)	47.375	12.284		63.251 *	12.910
SSP 1RM (kg)	23.839	6.773		30.491	8.539
SDL 1RM (kg)	52.667	10.234		70.811 *	14.776
Depression (BDI)	12.375	10.941		2.375 *	3.583
Anxiety (BAI)	16.250	17.670		4.875	5.490

* Statistically significant post-training change at *p* ≤ 0.05.

**Table 3:** The number of participants in each range of BDI scores at baseline and after the intervention.

BDI Score Ranges	Pre-Training Number of Participants in Each Range	Post-Training Number of Participants in Each Range
1–5	2	7
6–10	3	0
11–16	1	1
17–20	0	0
21–30	1	0
31–40	1	0
over 40	0	0

**Table 4:** The number of participants in each range of BAI scores at baseline and after the intervention.

BAI Score Ranges	Pre-Training Number of Participants in Each Range	Post-Training Number of Participants in Each Range
1–5	2	5
6–10	3	1
11–16	1	2
17–20	0	0
21–30	1	0
31–40	0	0
over 40	1	0

## Data Availability

The datasets presented in this article are not readily available as they are sensitive and contain information protected by HIPPA. Anonymized datasets are available upon request.
